# Two new species of the genus *Diostracus* Loew from Tibet, with a key to the Himalayan fauna (Diptera, Dolichopodidae)

**DOI:** 10.3897/zookeys.488.8919

**Published:** 2015-03-19

**Authors:** Ning Wang, Baohai Wang, Ding Yang

**Affiliations:** 1 Department of Entomology, College of Agronomy and Biotechnology, China Agricultural University, Beijing 100193; 2 Institute of Grassland Research, Chinese Academy of Agricultural Sciences, Hohhot, Inner Mongolia 010010, China; 3 Tibet Academy of Agricultural and Animal Husbandry Sciences, No.130 Jinzhu West Road, Lhasa, Tibet 850032, China

**Keywords:** Diptera, Dolichopodidae, *Diostracus*, new species, Tibet

## Abstract

Previously only one species of the genus *Diostracus* was known to occur in Tibet. Here the following two new species are added to the fauna of Tibet: *Diostracus acutatus* **sp. n.** and *Diostracus tibetensis* **sp. n.** Their relationships with similar species are discussed. A key to the species of *Diostracus* from the Himalayas is presented.

## Introduction

The genus *Diostracus* is a large genus in the subfamily Hydrophorinae and includes dolichopodids living on wet rocks and stones in mountain streams (Saigusa et al. 1997). It is distributed in the Holarctic and Oriental regions with 83 known species. Among them, three species are known to occur in the Nearctic region, 21 in the Palaearctic and 59 in the Oriental (Yang et al. 2006). The major references dealing with this genus are as follows: Takagi (1968, 1972), Negrobov (1980), Saigusa (1984), Saigusa et al. (1997), Yang (1998), Masunaga (2000) and Yang et al. (2011). Up to now, 23 species are recorded from China (Takagi 1968; Wei and Liu 1996; Yang 1998, 1999; Yang and Saigusa 2000; Zhang et al. 2003; Yang et al. 2011). The Chinese species were revised by Yang et al. (2011).

**Map 1.**
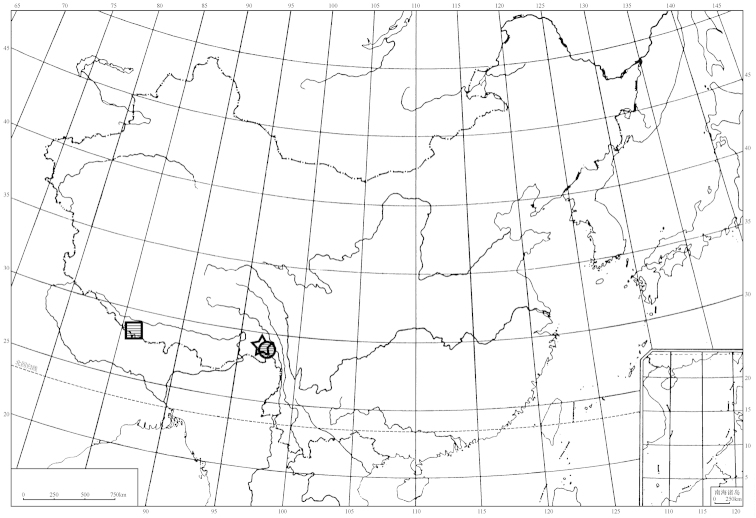
Distribution map of *Diostracus* in Tibet. ☆ *Diostracus acutatus* sp. n.; □ *Diostracus nebulosus* Takagi; ○ *Diostracus tibetensis* sp. n.

Only one species, *Diostracus nebulosus* Takagi, of the genus *Diostracus* was known to occur in Tibet (Yang et al. 2011). Here two new species are added to the fauna of Tibet, based on material collected by Dr. Zhaohui Pan and the junior author with Malaise traps (Map 1). Nine species groups for the species of *Diostracus* from the Himalayas were proposed by Saigusa (1984), and the three species from Tibet are placed within these groups. A key to the species of *Diostracus* from the Himalayas is presented.

## Material and methods

Type specimens are deposited in the Entomological Museum of China Agricultural University (CAU), Beijing. Morphological terminology generally follows McAlpine (1981) and Cumming and Wood (2009). The following abbreviations are used for bristles: acr–acrostichal, ad–anterodorsal, av–anteroventral, dc–dorsocentral, h–humeral, LI–fore leg, LII–mid leg, LIII–hind leg, npl–notopleural, oc–ocellar, pd–posterodorsal, ph–posthumeral, psa–postalar, pv–posteroventral, pvt–postvertical, sa–supraalar, sc–scutellar, vt–vertical.

## Taxonomy

### Key to the species (males) of *Diostracus* from the Himalayas

(modified from Saigusa 1984)

**Table:** 

1	Only posterior npl present, anterior npl absent (***unisetosus*-group**)	**2**
–	Anterior and posterior npl present	**4**
2	Discal cell with 1-2 accessory cellulae at anterodistal corner (Saigusa 1984, fig. 2; Saigusa 1995, fig. 3)	**3**
–	Discal cell without accessory cellulae at anterodistal corner (Saigusa 1984, fig. 1)	***Diostracus unisetosus* Saigusa**
3	6 dc; mid femur without spine-like av	***Diostracus nigrilineatus* Saigusa**
–	5 dc; mid femur with 3-5 spine-like av at basal 1/4	***Diostracus bisinuatus* Saigusa**
4	Empodium and pulvilli reduced into minute protuberance (***fenestratus*-group**)	**5**
–	At least empodium well developed, hair-like and ventrally ciliated	**14**
5	ph near transverse suture; vt absent (***fenestratus*-subgroup**)	6
–	ph near h; vt present	8
6	Mid and hind femora without long hairs and bristles; mid tibia long ciliated	**7**
–	Mid femur with thick clump of erect golden yellow pv hairs between basal 1/3 and 1/2, hind femur with row of erect av except basal and apical 1/4; mid tibia simple	***Diostracus ramulosus* Takagi**
7	Jet-black nodule of discal crossvein about 5 times as long as wide; 5 accessory cellulae formed at anterodistal corner of discal cell; cercus uniformly short yellow haired	***Diostracus reticulatus* Saigusa**
–	Jet-black nodule of discal crossvein about 2.6 times as long as wide; 3 accessory cellulae formed at anterodistal corner of discal cell; cercus with long golden hairs apically	***Diostracus fenestratus* Saigusa**
8	Discal crossvein strongly sinuate, S-shaped; anterodistal corner of discal cell with an accessory cellula (***pulchripennis*-subgroup**)	**9**
–	Discal crossvein nearly straight; anterodistal corner of discal cell without accessory cellula (***flex*-subgroup**)	**11**
9	Lateral portion of abdominal tergite 5 projected into an elongate process; mid tibia without row of erect fine posterior bristles	**10**
–	Lateral portion of abdominal tergite 5 expanded into a broad triangular lobe; mid tibia with row of erect fine posterior bristles	***Diostracus pretiosus* Saigusa**
10	Mid femur ventrally weakly raised subbasally, with modified hairs and bristles	***Diostracus emotoi* Saigusa**
–	Mid femur evenly flattened ventrally throughout and nearly bare	***Diostracus pulchripennis* Saigusa**
11	Wing shape normal, without finger-like lobe at posterior margin	**12**
–	Wing shape anomalous, with a finger-like lobe at posterior margin (Saigusa 1984, fig. 5)	***Diostracus pennilobatus* Saigusa**
12	Fore tarsomere 1 with an obtuse apicoventral corner, fore tarsomere 2 without finger-like process near extreme base (Saigusa 1984, figs 7–8)	**13**
–	Fore tarsomere 1 with a nearly acute apicoventral process, fore tarsomere 2 with a short finger-like ventral process near extreme base (figs 2, 5)	***Diostracus acutatus* sp. n.**
13	Fore tarsomere 1 with apicoventral corner rounded (Saigusa 1984, fig. 7); mid tibia ventrally not swollen near base	***Diostracus flexus* Saigusa**
–	Fore tarsomere 1 with apicoventral corner angulated (Saigusa 1984, fig. 8); mid tibia ventrally weakly swollen near base	***Diostracus nishidai* Saigusa**
14	Pulvilli atrophied, bare (***impulvillatus*-group**)	**15**
–	Pulvilli well developed, pad-like, pilose	**19**
15	Fore tarsomere 1 not furcate apically, fore tarsomere 2 shorter than tarsomere 1 (Saigusa 1984, figs 15–17)	**16**
–	Fore tarsomere 1 furcate apically, fore tarsomere 2 much longer than tasomere 1 (Saigusa 1984, fig. 18)	***Diostracus angustipalpis* Saigusa**
16	Wing without dark spot at discal crossvein; fore tarsomere 1 swollen apically (Saigusa 1984, figs 16–17)	**17**
–	Wing with a circular grayish spot at discal crossvein; fore tarsomere 1 not swollen apically (Saigusa 1984, fig. 15)	***Diostracus longiunguis* Saigusa**
17	Fore tarsomere 1 weakly dilated apically, 1.5 times thicker than its base (Saigusa 1984, fig. 17)	**18**
–	Fore tarsomere 1 strongly dilated apically, 3 times thicker than its base (Saigusa 1984, fig. 16)	***Diostracus chaetodactylus* Saigusa**
18	Cercus 3 times as long as wide, parallel-sided (Saigusa 1984, fig. 13)	***Diostracus impulvillatus* Saigusa**
–	Cercus about 1.5 times as long as wide, rather wide with narrow base (Saigusa 1984, fig. 14)	***Diostracus fulvispinatus* Saigusa**
19	Only 4 dc (***quadrisetosus*-group**)	**20**
–	5–6 dc	**28**
20	Leg mostly yellowish	**21**
–	Legs darkened, at most trochanters and knees tinged yellow	**23**
21	vt absent; fore tarsomere 1 without short erect av (Saigusa 1984, fig. 26)	**22**
–	vt present; fore tarsomere 1 with row of short erect av (Saigusa 1984, fig. 27)	***Diostracus parvus* Saigusa**
22	Anterior npl absent	***Diostracus janssonorum* Saigusa**
–	Anterior npl present	***Diostracus simplicipes* Saigusa**
23	Fore coxa subapically raised at anterior surface	**24**
–	Fore coxa normal	**25**
24	Fore femur not strongly swollen basally (Saigusa 1984, fig. 21); fore tarsomere 1 nearly straight	***Diostracus auripalpis* Saigusa**
–	Fore femur strongly swollen basally (Saigusa 1984, fig. 22); fore tarsomere 1 distinctly sinuate	***Diostracus femoratus* Saigusa**
25	Face without grayish median stripe	**26**
–	Face with a narrow grayish median stripe	***Diostracus makiharai* Saigusa**
26	R_4+5_ distinctly thicker than R_2+3_; palpus at most 0.9 times as long as eye height	**27**
–	R_4+5_ thinner than R_2+3_; palpus 1.1–1.2 times as long as eye height	***Diostracus magnipalpis* Saigusa**
27	Palpus enlarged, about 3/4 as long as eye height (Saigusa 1984, fig. 20)	***Diostracus aurifer* Saigusa**
–	Palpus not enlarged, about 1/3 as long as eye height	***Diostracus quadrisetosus* Saigusa**
28	Lower postocular bristles including posteroventral hairs on head yellow	**29**
–	Hairs and bristles on head wholly black (***nigripilosus*-group**)	***Diostracus nigripilosus* Saigusa**
29	First flagellomere triangular; arista subbasal	**30**
–	First flagellomere not triangular; arista apical, subapical or dorsal	**31**
30	vt much shorter and thinner than pvt; wing broadly darkened along veins; fore tarsomere 1 slender (Saigusa 1984, fig. 28) (***umbrinervis*-group**)	***Diostracus umbrinervis* Saigusa**
–	vt almost as strong as pvt; wing not broadly darkened along veins; fore tarsomere 1 distinctly thickened apically (Saigusa 1984, fig. 29) **(*tangalensis*-group)**	***Diostracus tangalensis* Saigusa**
31	Discal crossvein straight, without jet-black nodule or stripe (***nebulosus*-group**)	**32**
–	Discal crossvein bent, with a jet-black nodule or stripe	**33**
32	Mid and hind femora without ventral bristles; cercus long finger-like in lateral view (Saigusa 1984, fig. 4A)	***Diostracus burmanicus* Saigusa**
–	Mid and hind femora with ventral bristles; cercus short, subtriangular in lateral view (Yang et al. 2011, fig. 199d)	***Diostracus nebulosus* Takagi**
33	5 dc	**34**
–	At least 6 dc **(*unipunctatus*-group)**	**35**
34	M without dark cloud, discal crossvein with narrow jet-black nodule (Saigusa 1984, fig. 31)	***Diostracus alticola* Saigusa**
–	M with a dark cloud, discal crossvein with rounded jet-black nodule (Saigusa 1984, fig. 30)	***Diostracus shimai* Saigusa**
35	First flagellomere short circular, nearly as long as wide (Saigusa 1984, figs 38–39)	**36**
–	First flagellomere distinctly or strongly elongated, distinctly longer than wide (Saigusa 1984, figs 32–35)	**37**
36	Mid femur without very long yellow ventral bristles at base; first flagellomere with somewhat tapering apex (Saigusa 1984, fig. 39)	***Diostracus unipunctatus* Saigusa**
–	Mid femur with several very long yellow ventral bristles at base (Saigusa 1984, fig. 45); first flagellomere with wide apex (Saigusa 1984, fig. 38)	***Diostracus rotundicornis* Saigusa**
37	First flagellomere 1 apically strongly narrowed, not trapezoid	**38**
–	First flagellomere 1 nearly trapezoid	**39**
38	Arista nearly apical (Saigusa 1984, fig. 32); scutellum with strong sc	***Diostracus nepalensis* Saigusa**
–	Arista subapical (Saigusa 1984, fig. 33); scutellum without strong sc	***Diostracus gymnoscutellatus* Saigusa**
39	First flagellomere much elongated, about 2 times longer than wide (Saigusa 1984, figs 34, 36)	**40**
–	First flagellomere 1.3–1.5 times longer than wide (Saigusa 1984, figs 35, 37)	**41**
40	Arista located at dorsoapical corner of first flagellomere (Saigusa 1984, fig. 34)	***Diostracus auripilosus* Saigusa**
–	Arista dorsal (Saigusa 1984, fig. 36)	***Diostracus longicornis* Saigusa**
41	Arista subapical (Fig. 9; Saigusa 1984, fig. 35); vt distinctly longer than diameter of lateral ocellus	**42**
–	Arista dorsal; vt extremely reduced, as long as diameter of lateral ocellus	***Diostracus malaisei* Saigusa**
42	vt as strong as pvt; first flagellomere 1.5 times longer than wide (Saigusa 1984, fig. 35); abdominal tergite 5 with long yellow lateral hairs	***Diostracus parvipunctatus* Saigusa**
–	vt shorter and weaker than pvt; first flagellomere 1.3 times longer than wide (Fig. 9); abdominal tergite 5 with short lateral hairs	***Diostracus tibetensis* sp. n.**

### Diostracus acutatus sp. n.

Taxon classificationAnimaliaDipteraDolichopodidae

http://zoobank.org/A99B56FA-C496-4F1B-B60A-192FF11E0622

Figs 1–3 , 4–6

#### Diagnosis.

vt rather short, 0.5 times as long as oc. First flagellomere somewhat triangular, 1.5 times longer than wide; arista apical (Fig. 4). Fore tarsomere 1 distinctly shortened, thickened, concave ventrally, and with a nearly acute apicoventral process; tarsomere 2 basally bent, concave ventrally, and with a short finger-like ventral process near extreme base (Figs 2, 5). Crossvein m-cu much elongated, strongly bent (Fig. 3).

**Figures 1–3.**
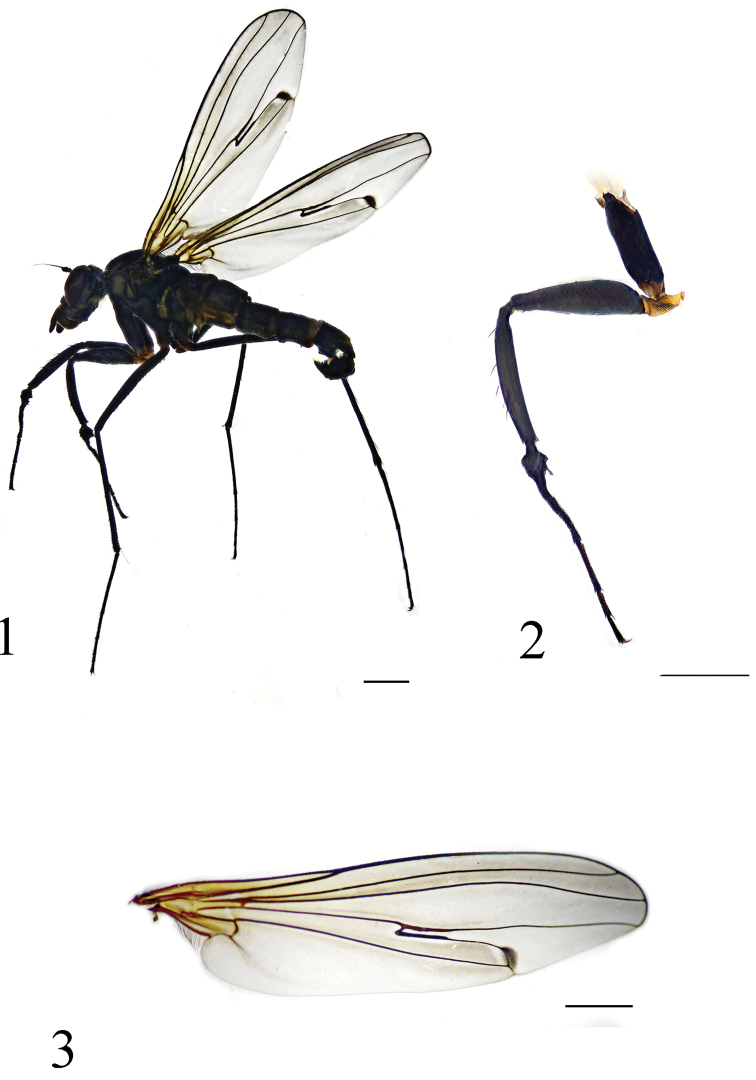
*Diostracus acutatus* sp. n. (male). **1** adult, lateral view **2** fore leg, posterior view **3** wing. Scale bar = 1 mm.

**Figures 4–6.**
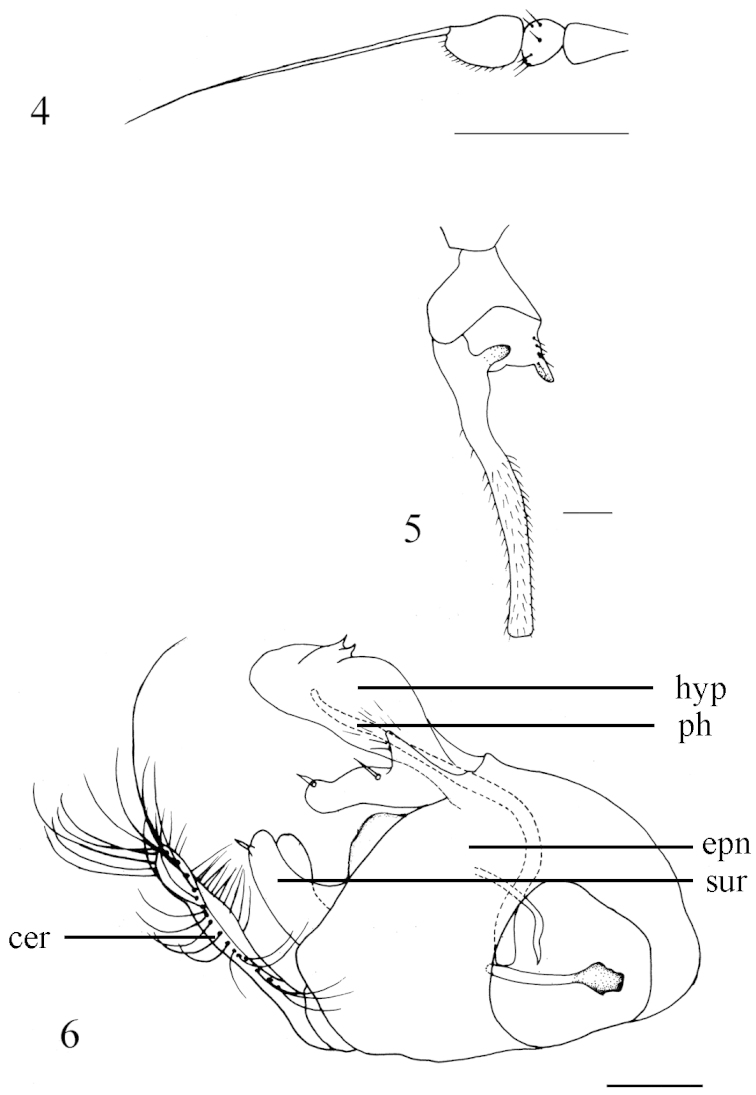
*Diostracus acutatus* sp. n. (male). **4** antenna, lateral view **5** fore tarsomeres 1–2, posterior view **6** genitalia, lateral view. Abbreviations: cer = cercus; epn = epandrium; hyp = hypandrium; sur = surstylus; ph = phallus. Scale bar = 0.2 mm.

#### Description.

Male. Body length 8.0 mm; wing length 8.5 mm.

Head metallic green with pale gray pollen. Eyes widely separated; face widened towards clypeus. Hairs and bristles on head black; lower postocular bristles including posteroventral hairs pale, mostly very long. Ocellar tubercle distinct, with pair of long strong oc, without posterior hairs; vt rather short, 0.5 times as long as oc, nearly as long as pvt. Antenna (Fig. 4) black; scape without any dorsal hairs; first flagellomere subtriangular, 1.5 times longer than wide; arista apical, 4 times as long as first flagellomere, nearly bare. Proboscis blackish with pale hairs; palpus lobate, 3.5 times as long as broad, produced far beyond apex of proboscis, blackish with a purple luster, and with black hairs.

Thorax metallic green with pale gray pollen; mesoscutum with two pairs of dark brown longitudinal spots (middle pair strip-like). Hairs and bristles on thorax black; 6 mostly hair-like dc except posteriormost 1 dc longest and thick; acr absent; 1 h and 1 very short hair, 1 ph, 2 npl, 1 sa, 1 psa; scutellum with pair of long sc. Propleuron with short pale hairs on upper portion and long pale hairs on lower portion. Legs nearly entirely black except fore trochanter dark yellow; claws well developed, empodium and pulvilli reduced. Fore trochanter elongated, with hook-like posterior process (Fig. 2). Fore femur distinctly thickened (Fig. 2). Mid femur slightly bent, somewhat flattened dorsoventrally. Fore tibia slightly thickened, weakly curved (Fig. 2). Fore tarsomere 1 distinctly shortened, thickened, concave ventrally, and with a nearly acute apicoventral process; tarsomere 2 basally bent, concave ventrally, and with a short finger-like ventral process near extreme base (Figs 2, 5). Hairs and bristles on legs black except those on coxae pale; fore coxa with group of long pale anterior hairs at base; hind coxa apically with 2 brownish anterior bristles. All femora somewhat bare ventrally, with only very short, sparse pale ventral hairs, except fore femur with 3 distinct pale av hairs basally. Mid femur with 3 anterior bristles on apical 1/3. Fore tibia with 4–5 ad and 1 posterior bristle at apical 1/3. Mid tibia with 3 pd, and with very long posterior hairs on apical 1/5 somewhat curved; apically with 1 short spine-like av. Hind tibia with 5 ad and 4 pd; apically with 1 ad. Relative lengths of tibia and five tarsomeres: LI 3.7: 0.7: 2.2: 1.35: 0.7: 0.75; LII 7.1: 3.7: 1.4: 0.9: 0.45: 0.65; LIII 7.6: 3.6: 2.1: 1.1: 0.5: 0.7. Wing (Fig. 3) hyaline, indistinctly tinged grayish; veins dark brown, R_4+5_ and M convergent apically; crossvein m-cu much elongated, strongly bent, margined with black on long anterior portion, and with blackish spot at short posterior portion. Squama brown with pale hairs. Halter brown.

Abdomen distinctly longer than head and thorax combined, metallic green with pale gray pollen. Abdomen with pale pubescence. Tergite 5 with lateral portion slightly extended downward. Sternite 1 with a nearly acute process at middle; sternite 4 medially with an obtuse anterior process and 2 short thin, contiguous posterior processes bearing bundle of brown hairs. Hypandrium not distinctly swollen.

Male genitalia (Fig. 6): Epandrium slightly longer than wide. Epandrial lobe short thick, finger-like, weakly bent, with an acute basal process; 1 slightly long bristle present at middle and 1 short thick bristle at tip. Surstylus short thick, apically furcated, with 1 very short apical denticle bearing 1 very short spine-like bristle. Hypandrium short thick, apically with a shallow, V-shaped apical incision, subapically with 2 small acute processes. Cercus slightly bent, nearly finger-like in lateral view, with long dark yellow hairs.

Female. Unknown.

#### Type material.

Holotype: male, China: Tibet, Nyingchi (N29°38'18", E94°21'46"), Sejilashan Mountain, Zhongshan Station, 4200 m, 20.VI.–10.VII. 2014, Malaise trap, leg. Baohai Wang and Zhaohui Pan (CAU).

#### Distribution.

China (Tibet).

#### Remarks.

The new species belongs to the *flexus*-subgroup of the *fenestratus*-group. It may be separated from *Diostracus flexus* Takagi and *Diostracus nishidai* Saigusa from Nepal by the fore tarsomere 1 with a nearly acute apicoventral process and fore tarsomere 2 with a short finger-like ventral process near the extreme base (Figs 2, 5). In *Diostracus flexus* and *Diostracus nishidai*, the fore tarsomere 1 has an obtuse apicoventral corner, and the fore tarsomere 2 has no finger-like process near the extreme base (Saigusa 1984, figs 7–8).

#### Etymology.

The specific name refers to the fore tarsomere 1 with a nearly acute apicoventral process.

### Diostracus nebulosus

Taxon classificationAnimaliaDipteraDolichopodidae

Takagi, 1972

#### Diagnosis.

First flagellomere (Yang et al. 2011, fig. 199b) 1.3 times longer than wide, obtuse apically; arista dorsal. Wing (Yang et al. 2011, fig. 199a) with an obscure spot at anteroapical corner of discal cell; crossvein m-cu straight. Male cercus (Yang et al. 2011, fig. 199d) short, subtriangular.

#### Distribution.

China (Tibet), Nepal.

#### Remarks.

This species belongs to the *nebulosus*-group.

### Diostracus tibetensis sp. n.

Taxon classificationAnimaliaDipteraDolichopodidae

http://zoobank.org/227A5CD2-DAE4-48A8-88FF-036F31E41D3F

Figs 7–8 , 9–10

#### Diagnosis.

vt rather short and weak, 0.4 times as long as oc. First flagellomere somewhat quadrate, 1.3 times longer than wide; arista subapical (Fig. 9). Wing (Fig. 8) hyaline; crossvein m-cu medially distinctly bent with small round black nodule located at middle of crossvein. Fore coxa with bundle of short dense black anterior hairs bristle-like at extreme tip. Mid and hind femora with very long pale ventral hairs (longest ones about 3 times as long as femur thickness). Abdominal tergites 4–5 with lateral portion slightly extended downwards, only tergite 4 with very long lateral hairs.

**Figures 7–8.**
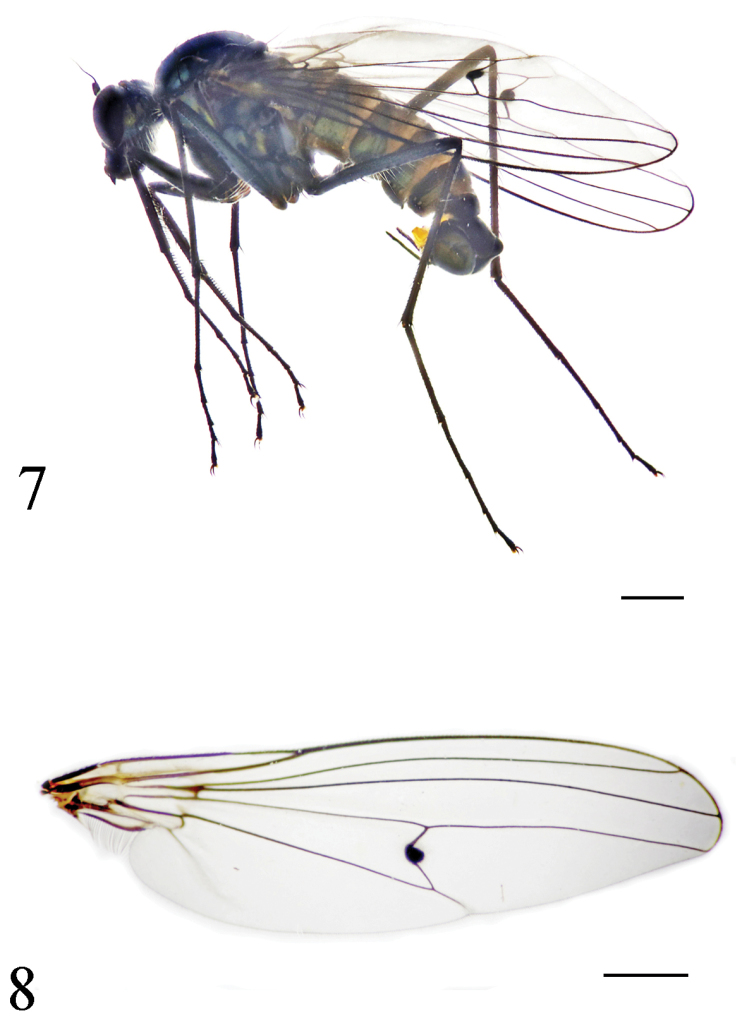
*Diostracus tibetensis* sp. n. (male). **7** adult, lateral view **8** wing. Scale bar = 1 mm.

**Figures 9–10.**
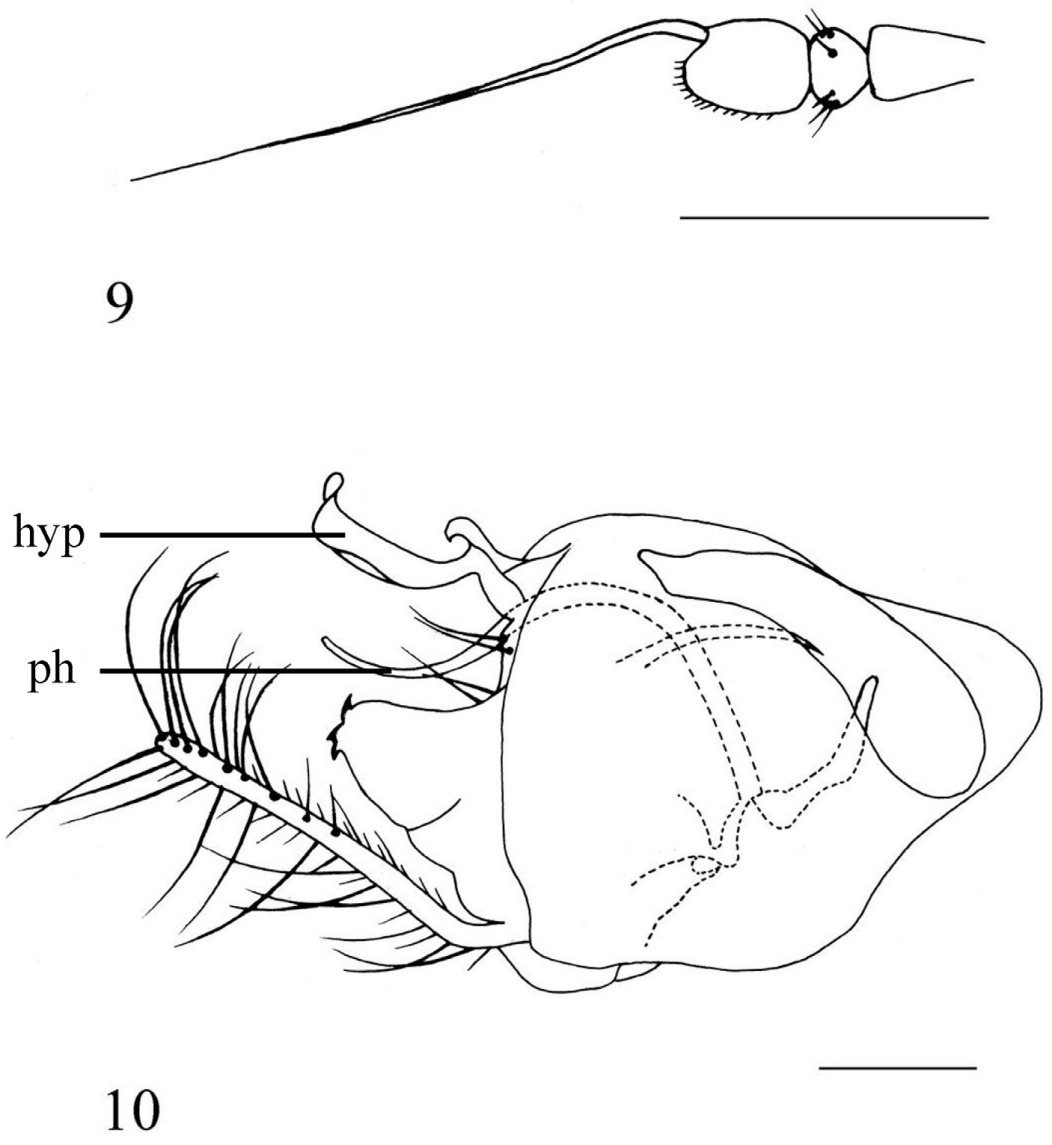
*Diostracus tibetensis* sp. n. (male). **9** antenna, lateral view **10** genitalia, lateral view. Scale bar = 0.2 mm.

#### Description.

Male. Body length 6.4 mm; wing length 7.6 mm.

Head metallic green with pale gray pollen. Eyes widely separated; face widened towards clypeus. Hairs and bristles on head black; lower postocular bristles including posteroventral hairs pale, mostly very long. Ocellar tubercle distinct, with pair of strong oc, without posterior hairs; vt rather short and weak, 0.4 times as long as oc, 0.7 times as long as pvt. Antenna (Fig. 9) black; scape without any dorsal hairs; first flagellomere short, somewhat quadrate, 1.3 times longer than wide; arista subapical, 3.9 times as long as first flagellomere, nearly bare. Proboscis blackish with pale hairs; palpus lobate, smoky black with black hairs.

Thorax metallic green with pale gray pollen; mesoscutum with two pairs of dark brown longitudinal spots (middle pair strip-like). Hairs and bristles on thorax black; 6 slightly long dc, posteriormost dc longest; acr bristles absent; 1 h and 1 short bristle, 1 ph, 2 npl, 1 sa, 1 psa; scutellum with pair of long sc and 4 very short marginal hairs (2 hairs between 2 sc). Propleuron with short pale hairs on upper portion and mostly long pale hairs on lower portion. Legs entirely black; claws well developed, empodium and pulvilli distinct. Hairs and bristles on legs black except those on coxae pale; fore coxa with bundle of short dense black anterior hairs bristle-like at extreme tip; hind coxa apically with 4 long blackish anterior hairs bristle-like. Mid and hind femora with some pale ventral hairs. Fore femur with two rows of black ventral bristles (longest ones slightly shorter than femur thickness), and with 3 long posterior bristles at extreme base. Mid femur basally with nearly two close rows of long pale ventral hairs (longest ones about 3 times as long as femur thickness), subbasally with 4 black short thick av. Hind femur with about two close rows of long pale ventral hairs (longest ones about 3 times as long as femur thickness) and with 5 black short thick av. Fore tibia with 4 ad and 4 pv on apical half; apically with 3 bristles. Mid tibia with 3 ad and 2 pd; apically with 3 bristles. Hind tibia with 4 ad, 5 pd, 3 av and 6 pv; apically with 3 bristles. Fore tarsomere 1 with row of short dense erect av spines and one row of dense thin pv (longer than av). Relative lengths of tibia and five tarsomeres: LI 3.3: 1.6: 1.6: 0.8: 0.55: 0.7; LII 5.8: 2.9: 1.1: 0.75: 0.5: 0.75; LIII 7.1: 3.2: 1.9: 1.2: 0.6: 0.8. Wing (Fig. 8) hyaline; veins dark brown, R_4+5_ and M convergent apically; crossvein m-cu medially distinctly bent with small round black nodule located at middle of crossvein. Squama brown with pale hairs. Halter brown to dark brown.

Abdomen rather short, nearly as long as head and thorax combined, metallic green with pale gray pollen. Abdomen with pale pubescence except dorsum with some black hairs at middle. Tergites 4 distinctly and tergite 5 weakly with lateral portion extended downward; lateral portion of tergite 4 with very long hairs apically bent, slightly shorter than those on sternite 3, but lateral portion of tergite 5 only with short hairs.

Male genitalia (Fig. 10): Epandrium relatively short, slightly longer than wide. Epandrial lobe weak, with 2 long bristles. Surstylus enlarged, with three acute denticles at apical margin. Hypandrium narrowed, bent; apically with a shallow, V-shaped apical incision and lateral lobe curled; basally with a hook-like process. Cercus straight, long finger-like, with long yellow hairs.

Female. Unknown.

#### Type material.

Holotype: male, China: Tibet, Nyingchi (N29°38'18", E94°21'46"), Sejilashan Mountain, Zhongshan Station, 4200 m, 20.VI.-10.VII. 2014, Malaise trap, leg. Baohai Wang and Zhaohui Pan (CAU).

#### Distribution.

China (Tibet).

#### Remarks.

The new species belongs to the *unipunctatus*-group. It is somewhat similar to *Diostracus parvipunctatus* Saigusa from Nepal in the shape of the first flagellomere and fore and mid femora with long ventral hairs, but may be separated from the latter in the following points: vt is shorter and weaker than pvt; the first flagellomere is shorter, 1.3 times longer than wide, and the abdominal tergite 5 has the short lateral hairs. In *Diostracus parvipunctatus*, vt is as strong as pvt or stronger; the first flagellomere is 1.5 times longer than wide (Saigusa 1984, fig. 35), and the abdominal tergites 4–5 has the long yellow lateral hairs (Saigusa 1984).

#### Etymology.

The specific name refers to the type locality Tibet.

## Supplementary Material

XML Treatment for Diostracus acutatus

XML Treatment for Diostracus nebulosus

XML Treatment for Diostracus tibetensis
